# Intrinsic Sources and Functional Impacts of Asymmetry at Electrical Synapses

**DOI:** 10.1523/ENEURO.0469-21.2022

**Published:** 2022-03-11

**Authors:** Austin J. Mendoza, Julie S. Haas

**Affiliations:** Department of Biological Sciences, Lehigh University, Bethlehem, PA 18015

**Keywords:** asymmetry, computational model, electrical synapse, gap junction, rectification

## Abstract

Electrical synapses couple inhibitory neurons across the brain, underlying a variety of functions that are modifiable by activity. Despite recent advances, many functions and contributions of electrical synapses within neural circuitry remain underappreciated. Among these are the sources and impacts of electrical synapse asymmetry. Using multi-compartmental models of neurons coupled through dendritic electrical synapses, we investigated intrinsic factors that contribute to effective synaptic asymmetry and that result in modulation of spike timing and synchrony between coupled cells. We show that electrical synapse location along a dendrite, input resistance, internal dendritic resistance, or directional conduction of the electrical synapse itself each alter asymmetry as measured by coupling between cell somas. Conversely, we note that asymmetrical gap junction (GJ) conductance can be masked by each of these properties. Furthermore, we show that asymmetry modulates spike timing and latency of coupled cells by up to tens of milliseconds, depending on direction of conduction or dendritic location of the electrical synapse. Coordination of rhythmic activity between two cells also depends on asymmetry. These simulations illustrate that causes of asymmetry are diverse, may not be apparent in somatic measurements of electrical coupling, influence dendritic processing, and produce a variety of outcomes on spiking and synchrony of coupled cells. Our findings highlight aspects of electrical synapses that should always be included in experimental demonstrations of coupling, and when assembling simulated networks containing electrical synapses.

## Significance Statement

Asymmetry, or unequal transmission of current between two coupled neurons, is a property of electrical synapses often noted but seldom explored. Here, we show that multiple intrinsic factors can either produce, or mask, asymmetry. Spike timing and rhythmic synchrony are both affected by asymmetric connections between neurons. These results highlight important consequences of asymmetry that are likely to be recapitulated within coupled networks throughout the brain.

## Introduction

Electrical synapses represent a major form of communication between neurons across neuronal tissue, with many impacts that have not been extensively explored. Asymmetry of transmission, is a frequently noted aspect of electrical synapses: it is the property of unequal transmission of electrical signals between two neurons, and ranges in effect from minor to complete. Electrical synapses have been well studied in invertebrates, where evidence of asymmetry comes from species including crayfish (Furshpan and Potter, 1959), *Drosophila* giant fibers (Phelan et al., 2008), lobster stomatogastric ganglion (Johnson et al., 1993), and the *Caenorhabditis elegans* escape circuit (Liu et al., 2017; Shui et al., 2020). In invertebrate systems, asymmetry varies widely, with some synapses displaying full rectification. In contrast, asymmetry at synapses between mammalian neurons is often more modest. Demonstrations of electrical synapse asymmetry are numerous throughout the mammalian brain, including retina (Veruki and Hartveit, 2002), cortex (Galarreta and Hestrin, 2002), inferior olive (Devor and Yarom, 2002), dorsal cochlear nucleus (Apostolides and Trussell, 2013), mesencephalic trigeminal nucleus (Curti et al., 2012), cerebellar Golgi cells (Szoboszlay et al., 2016) and molecular layer interneurons (Mann-Metzer and Yarom, 1999; Alcami and Marty, 2013), and the thalamic reticular nucleus (TRN; Haas et al., 2011; Sevetson and Haas, 2015; Zolnik and Connors, 2016). Recent results show that asymmetry can be modified during the activity that results in electrical synapse plasticity (Haas et al., 2011; Fricker et al., 2021), indicating that it is a dynamic property that is under activity-dependent regulation.

Asymmetry of electrical transmission can in principle result from a wide variety of influences. It has been well established in non-mammalian systems that directional differences in conductance between two coupled cells can result from heteromeric channels or heterotypic gap junction (GJ) plaques that coupled membranes (Bukauskas et al., 1995; Rash et al., 2013), or from differences in hemichannel protein scaffolding (Marsh et al., 2017). Hemichannel differences resulting in asymmetry have been demonstrated in HeLa cells expressing connexin isoforms (Bukauskas et al., 1995) and at the mixed synapse onto Mauthner cells in goldfish (Rash et al., 2013). Connexin-sourced asymmetry was thought to be unlikely for neuronal mammal synapses, as connexin36 does not oligomerize or dock with other connexins (Teubner et al., 2000; Li et al., 2004), and in expression systems appears to form perfectly symmetric synapses (Srinivas et al., 1999). However, residual coupling has been noted between TRN neurons in connexin36 knock-out mice, and that coupling was more asymmetrical (Zolnik and Connors, 2016), indicating a possible physiological source of synaptic asymmetry in mammalian neuronal systems. Large gradients of Mg^2+^ concentration produce asymmetric signaling for neuronal synapses (Palacios-Prado et al., 2013), and gating properties of connexin channels produce asymmetry in computational models (Snipas et al., 2017). Cable properties of coupled dendrites affect voltage transmission (Nadim and Golowasch, 2006), indicating that differences in dendritic diameter may produce asymmetric coupling. Intrinsic differences between coupled neurons, such as differences in input resistance (Bennett, 1966; Mann-Metzer and Yarom, 1999; Veruki and Hartveit, 2002; Fortier, 2010) or leak conductances (Alcami and Marty, 2013), have long been mentioned as a straightforward reason that one might observe asymmetry in coupling coefficients. For TRN synapses, asymmetry remains even after computing estimates of conductance that should in principle minimize contributions of input resistance (Haas et al., 2011; Sevetson and Haas, 2015). And while many reports of electrical synapses across the mammalian brain include asymmetry in their measurements, some reports do not note it, or only note that in their observations, synapses were symmetrical as expected. In all, asymmetry and its sources remain underappreciated at mammalian electrical synapses.

Beyond observations, the functional consequences of electrical synapse asymmetry on neural activity are not robustly understood. Electrical synapses have been widely shown to contribute toward synchrony of rhythmic activity in neuronal networks in both experiments (Marder, 1998; Draguhn et al., 1998; Galarreta and Hestrin, 1999; Gibson et al., 1999; Mann-Metzer and Yarom, 1999; Tamás et al., 2000; Hormuzdi et al., 2001; Landisman et al., 2002; Blatow et al., 2003; Bennett and Zukin, 2004; Long, 2004; Christie et al., 2005; Long et al., 2005; Vervaeke et al., 2010) and in computational models (Kepler et al., 1990; Sherman and Rinzel, 1992; Destexhe et al., 1996; Manor et al., 1997; Skinner et al., 1999; Chow and Kopell, 2000; Lewis and Rinzel, 2003; Whittington and Traub, 2003; Kopell and Ermentrout, 2004; Nomura et al., 2004; Saraga and Skinner, 2004; Pfeuty et al., 2005; Traub et al., 2005; O’Connor et al., 2012; Gutierrez et al., 2013; Pernelle et al., 2018), and oscillations are more robust when asymmetrical electrical synapses are included (Gutierrez and Marder, 2013). Rectification at the LP-PY mixed synapse is a key component of coordinating the pyloric circuit of the spiny lobster (Mamiya et al., 2003). In non-rhythmic settings, strong asymmetry can produce nearly unidirectional communication that serves to reliably excite one coupled cell, as is the case with the club endings onto Mauthner cells in goldfish (Rash et al., 2013), and dorsal cochlear nucleus (Apostolides and Trussell, 2013). Electrical synapses modulate individual spike times in coupled neighbors in TRN by up to tens of milliseconds (Haas, 2015; Sevetson and Haas, 2015), and asymmetric coupling can add to that modulation, even reversing firing order between two coupled cells that receive closely-timed inputs (Sevetson and Haas, 2015). In a model thalamocortical circuit, coupling between feedback inhibitory neurons enhances discrimination of inputs sent to cortex by relay cells (Pham and Haas, 2018). In a canonical model circuit with feedforward inhibition, electrical synapses enhance subthreshold integration in principal cells (Pham and Haas, 2019). In a toadfish vocal circuit, electrical coupling between feedforward inhibitory neurons enhances synchrony and temporal precision (Chagnaud et al., 2021) and a similar effect occurs for cerebellar basket cells (Alcami, 2018; Hoehne et al., 2020). These are some of the functions that could be altered by asymmetry. While a few models have included electrical synapses in morphologically extended cells (Saraga and Skinner, 2004; Nadim and Golowasch, 2006; Amsalem et al., 2016), the sources or functions of asymmetrical synapses have not yet been explored in that context.

Here, we used compartmental models of coupled TRN neurons to investigate and compare how a variety of fundamental neuronal properties could each contribute to electrical synapse asymmetry, including synapse location, strength, direction of conductance, dendritic geometry, and input resistance. We show that, as predicted, a variety of these factors can produce effective differences in coupling coefficients as observed between somas. We then demonstrate that conversely, these same properties can mask asymmetric conductance of electrical synapses, resulting in apparently equal coupling as measured between somas. Together, these results underline that asymmetric transmission and impacts are likely to occur more widely than previously considered. Finally, we show that asymmetry regulates spike timing and, unexpectedly, the form of rhythmic coordination in coupled neurons. We conclude that electrical synapses between dendrites can exert locally powerful influence that is not readily apparent at the soma, highlighting the necessity of including electrical synapses in morphologically detailed models, circuits or connectomes.

## Materials and Methods

### Modelling

Models were built on those previously reported (Destexhe et al., 1996; Traub et al., 2005; Haas and Landisman, 2012; Pham and Haas, 2018, 2019). We use Hodgkin–Huxley formalism (Eq. 1) solved by a second order Runge–Kutta ODE solver in MATLAB version R2020b (MathWorks), simulations were run on an ASUS desktop PC with Intel i7-10700K CPU running Windows 10:

(1) $$\begin{array}{l} C_{m}\frac{dV_{i}}{dt}=G_{leak}\cdot \left(E_{leak}-V_{i}\right)+\underset{\begin{array}{c} ion\\ channels \end{array}}{\sum }G_{ion}(t)\cdot (E_{ion}-V_{i})\\ +\overset{j\neq i}{\underset{\begin{array}{c} chemical\\ synapses \end{array}}{\sum }}G_{syn}\left(t,t_{j}^{events}\right)\cdot \left(E_{syn}-V_{i}\right)+\overset{j\neq i}{\underset{\begin{array}{c} electrical\\ synapses \end{array}}{\sum }}G_{elec\;ji}\cdot (V_{j}-V_{i})\\ +\underset{\begin{array}{c} external\\ inputs \end{array}}{\sum }G_{syn}\left(t,t_{external}^{events}\right)\cdot (E_{syn}-V_{i})+\overset{j\neq i}{\underset{\begin{array}{c} coupled\\ compartments \end{array}}{\sum }}G_{internal\;ji}\cdot (V_{j}-V_{i}) \end{array}$$

The single compartment TRN cell model included the following ionic currents and maximal conductances: fast transient Na^+^ (Na_T_) 60.5 mS/cm^2^, K^+^ delayed rectifier (K_d_) 60 mS/cm^2^, K^+^ transient A (K_t_) 5 mS/cm^2^, slowly inactivating K^+^ (K_2_) 0.5 mS/cm^2^, slow anomalous rectifier (AR) 0.025 mS/cm^2^, and low threshold transient Ca^2+^ (Ca_T_) 0.75 mS/cm^2^. Reversal potentials were 50 mV for sodium, −100 mV for potassium, 125 mV for calcium, −40 mV for AR and −75 mV for leak. Capacitance was 1 μF/cm^2^ with leak of 0.1 mS/cm^2^. Three-compartment models were constructed consisting of one soma and two dendritic compartments, approximating the middle and distal regions of the dendrite. Compartments were connected by a static conductance G_internal_ of 0.35 mS/cm^2^ between distal and middle dendrites, and 0.4 mS/cm^2^ between middle dendrite and soma. Membrane capacitance was 1.2 μF/cm^2^. Maximal conductance for the compartmental model were: Na_T_ 60.5 mS/cm^2^, K_d_ 90 mS/cm^2^, K_t_ 5 mS/cm^2^, K_2_ 0.5 mS/cm^2^, AR 0.005 mS/cm^2^, and Ca_T_ 0.5 mS/cm^2^. Leak conductance was set at 0.1 mS/cm^2^ for soma compartments, and 0.035 mS/cm^2^ for dendrites, except when altering input resistance where leak conductance was scaled by 0.75–1.45 times, corresponding to ±25% change in input resistance. Dendritic compartments had lower Ca_T_ conductance of 0.15 mS/cm^2^. We removed the sodium current from dendrites, as TRN dendrites do not spike in recordings (Connelly et al., 2017). Electrical synapses were modeled as a static conductance G_elec_ (referred to as G_c_ in Results) applied to the voltage difference between the coupled compartments of the TRN cells. Asymmetry was implemented by varying G_elec_ for each cell. Excitatory synapses were AMPAergic with reversal potential of 0 mV with rise and fall time kinetics of 5 ms and 35 ms respectively.

### Analysis

Coupling coefficients were measured by injecting hyperpolarizing current into the soma of one cell (A) and measuring the resulting current deflection in the soma of the other cell (B) compared with baseline (cc_AB_ = ΔV_B_/ΔV_A_), matching experimental methodology. We used 500-ms-long square current injection and measured coupling in both directions between the cell pairs. The steady-state voltage during hyperpolarization was taken as the average voltage during the last 200 ms of stimulation. Coupling coefficient ratio was calculated as cc_12_/cc_21_.

To analyze latency modulation produced by electrical synapses we applied burst like EPSCs to distal dendrites of the model TRN cells and measured the time between onset of the first EPSC to the first action potential. Bursts consisted of 13 EPSCs of 1-μA amplitude with 5-ms interspike interval (ISI), 0.5-μA depolarizing current was applied to raise excitability of the cell model. Latency modulation was expressed as the difference in the change of latency between the two cells, compared with the latency of an uncoupled model cell. Synchrony was examined using single cell models driven to spike tonically, with one cell driven slightly higher, I1 = 0.575, I2 = 0.6 μA/cm^2^. Cross-correlations were taken for a 500-ms time window during stable tonic firing, spike trains were filtered with a 5-ms Hanning window. Time lag values from the peak of the cross correlations were taken to calculate phase difference between the two spike trains (phase difference = t_max lag_/ISI × 360). To examine the effect of asymmetry, coupling was constant in the cell 2–1 direction and scaled coupling in the 1–2 direction to obtain ratios between 0.3 and 3 times, the range observed from paired recordings at TRN.

### Code availability

The code/software described in the paper is freely available online at https://github.com/jhaaslab/Asymmetry. The code is also available as Extended Data 1.

10.1523/ENEURO.0469-21.2022.ed1 Extended Data 1 Asymmetry Project code. Download Extended Data 1, ZIP file.

## Results

**Figure 1.**
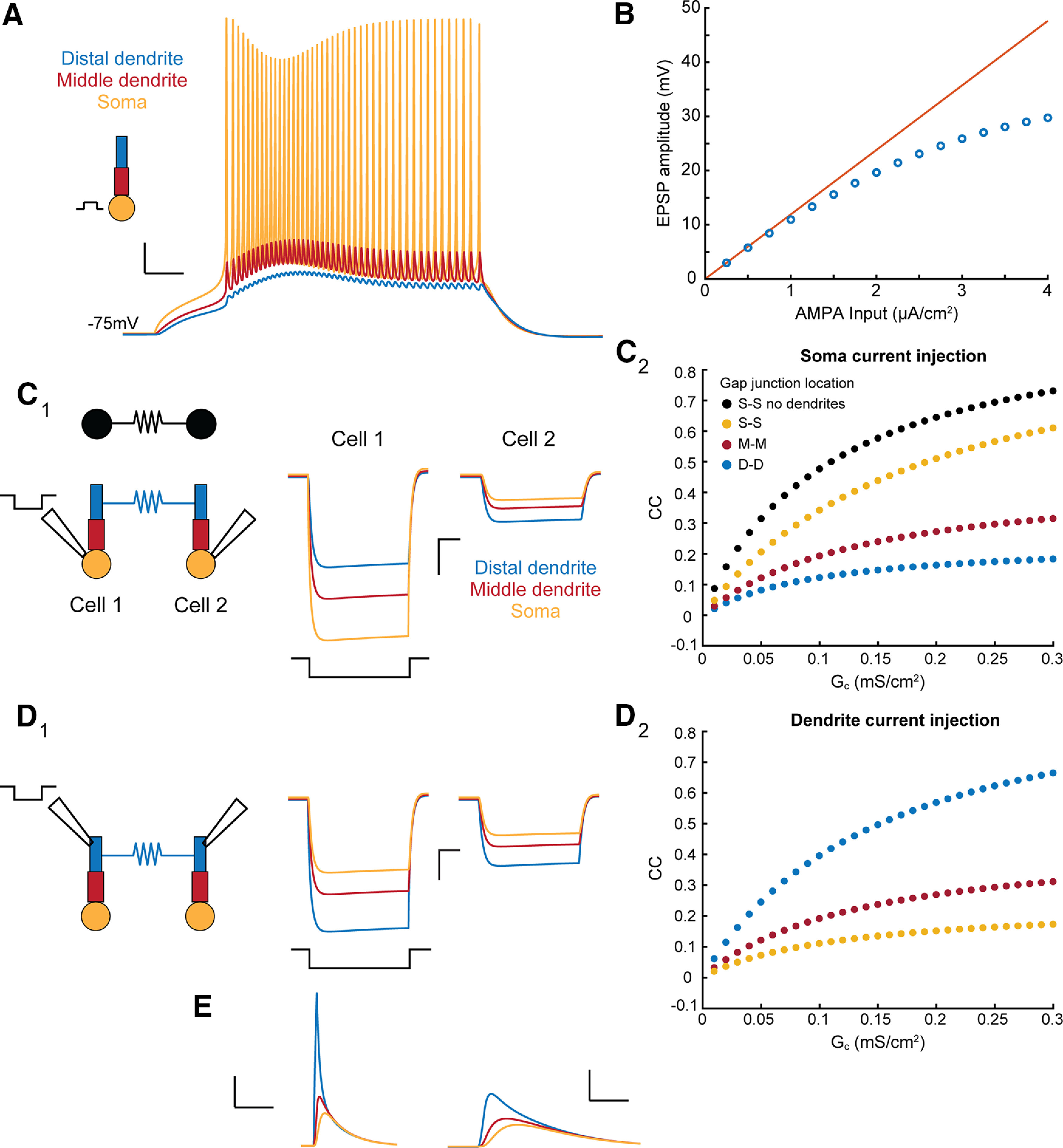
Compartmental TRN model and characterization of coupling responses for GJs at varied dendritic locations. ***A***, Schematic of compartmental TRN model and representative traces from each compartment in response to square depolarizing current injection at the soma compartment. Scale bars: 10 mV, 25 ms. ***B***, Postsynaptic response to an AMPAergic input delivered to distal dendrite. EPSP amplitudes were sublinear above 2 μA, due to lack of active conductances in dendrites. ***C_1_***, Schematic for electrically coupled models. Voltage traces in both cells result from a hyperpolarizing current injection into the soma of cell 1 (left traces) and transfer to cell 2 (right traces). Scale bars: 5 mV, 100 ms. ***C_2_***, Coupling coefficients (cc) measured between somatic compartments as in ***C_1_*** for GJs located between the somas (yellow), middle compartments (red), and distal dendrites (blue). An identical single-compartment model is shown for comparison (black). ***D_1_***, Schematic and voltage traces for coupling measured between dendrites after dendritic current injection. ***D_2_***, Coupling coefficients (cc) measured between dendritic compartments as in ***D_1_*** for GJs located between the somas (yellow), middle compartments (red), and distal dendrites (blue). ***E***, EPSP amplitudes for each compartment for distally applied EPSC to cell 1 (left traces; scale bars: 2 mV, 25 ms), transmitted to cell 2 across a distal electrical synapse (right traces; scale bars: 1 mV, 12.5 ms).

To address the impact of neuronal excitability and morphology on electrical synapse communication, we built a three-compartment TRN cell Hodgkin–Huxley model, including a T-type calcium conductance in addition to leak, sodium, and potassium conductances, based on those previously used (Destexhe et al., 1996; Traub et al., 2005; Pham and Haas, 2018, 2019). To validate the model’s dendritic responses, we used coupling between compartments that generated reasonable amplitudes of backpropagated signals (Fig. 1*A*), sublinear dendritic responses to AMPAergic current injections (Fig. 1*B*) that matched dendritic recordings from TRN cells (Connelly et al., 2017) and firing responses from our own recordings (Haas et al., 2011; Sevetson and Haas, 2015). We then added an electrical synapse between matched compartments of two identical TRN model cells and measured the coupling coefficients resulting from hyperpolarizing current applied to and measured at the somas (Fig. 1*C1*). We chose coupling conductance values to match coupling coefficients observed in recordings, cc between 0 and 0.3 (Haas et al., 2011). For these matched-compartment connections, electrical synapses produced higher coupling coefficients when synapses were closer to the soma, as the current between electrodes has a more direct path (Fig. 1*D1*). A single-compartmental model used for comparison (Fig. 1*C1,D1*, black) resulted in stronger coupling coefficients, as removing dendrites reduced leaks from the circuit. Similarly, when current is applied and coupling measurements are taken between distal dendrites (Fig. 1*C2*), coupling is stronger at dendritically located synapses but decreases as the synapse location approaches the soma (Fig. 1*D2*). This is a simple result of cable properties, but highlights the notion that electrical synapses can produce strong and effective coupling between dendritic compartments (Fig. 1*E*) that is not apparent from somatic measurements.

**Figure 2.**
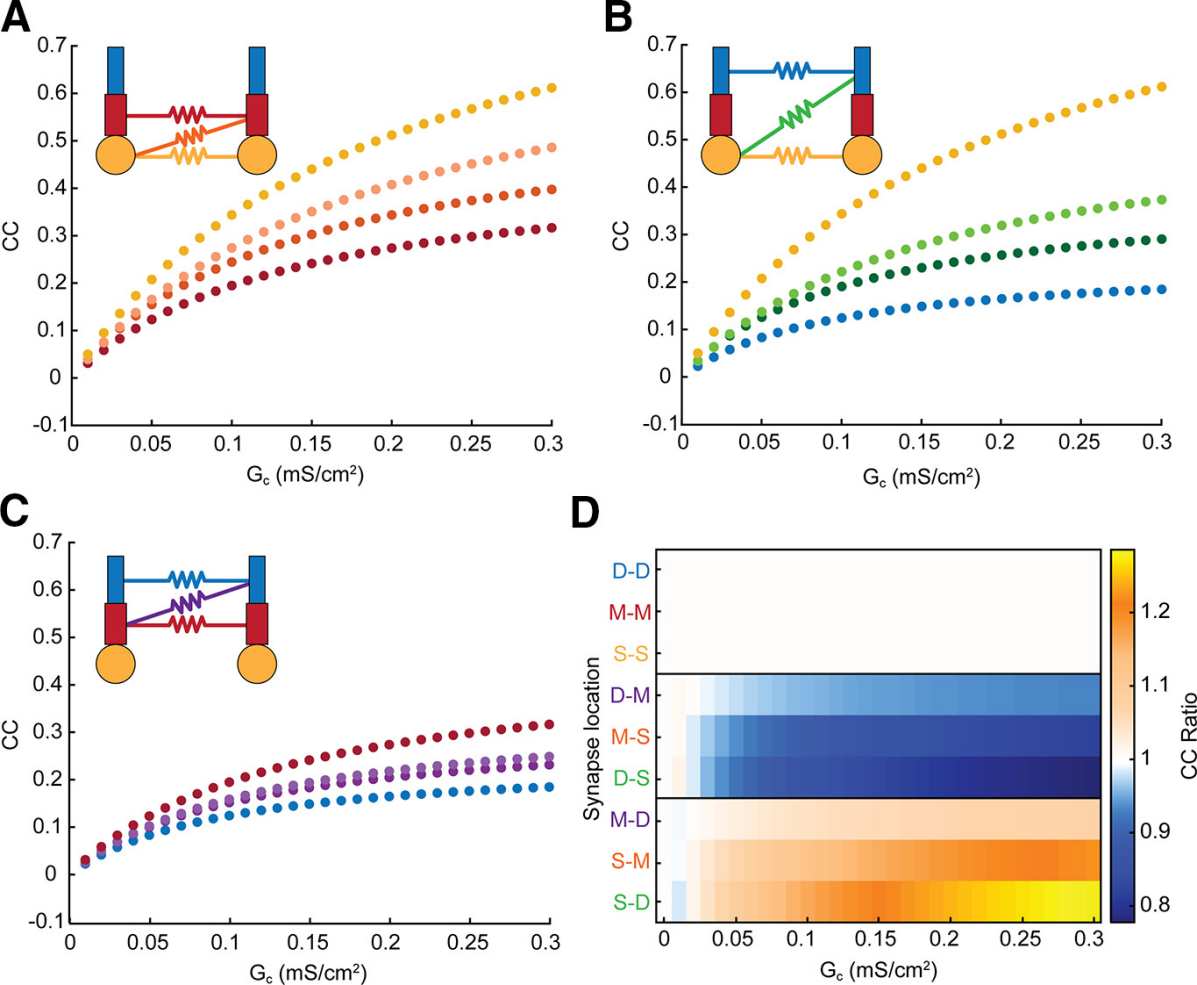
Electrical synapse location and strength contribute to coupling coefficient and asymmetry measured at the soma. ***A***, Coupling coefficients measured from cell 1 to cell 2 for all sets of somatic and middle-compartment electrical synapses. Soma-soma synapses are in yellow, middle-middle synapses are in red, and both types of soma-middle synapses are orange with opposite coupling (M-S) indicated by darker shaded datapoints. ***B***, As in ***A*** for somatic and distal synapse locations. ***C***, As in ***A*** for middle and distal synapse locations; note overall decrease in coupling for more-distant synapses. ***D***, Coupling coefficient ratio (cc_12_/cc_21_) for each synapse location and strength. Locations are grouped by effect; the top box shows GJs between matched compartments that are symmetric, as expected. In the middle box, the GJ was closer to the soma of cell 2, and thus cc_21_ was larger. In the bottom box, the GJ was closer to the soma of cell 1, and thus cc_12_ was larger. Asymmetry increased with difference between location of the GJ, and with proximity to the soma.

Next, we varied the location of electrical synapses between the dendritic compartments of each cell, and again measured coupling between somas of cell 1 and cell 2. In all cases, the coupling for mixed-location synapses was intermediate to the values obtained for connections between matched compartments (Fig. 2*A–C*). Interestingly, coupling values for pairs of compartment connections that one might initially expect to produce the same coupling, such as soma-middle (S-M) and middle-soma (M-S; Fig. 2*A*, orange dots), do not produce identical coupling coefficients. The source of asymmetry in this case is the differences in dendritic leaks that siphon soma-applied current from the GJ pathway. Specifically, for S-M connections, the dendritic load for soma-applied current comprises resistance from both M and D compartments and is larger than the remaining dendritic leak from a single D compartment when current is applied to the opposite soma. These differences lead to differences in the currents crossing the GJ when current is separately applied to each soma, and thus the coupling coefficients are asymmetric as measured between somas. Comparing Figure 2*A–C*, we note that this effect is strongest for connections closer to the soma, where the differences in dendrites distal to the electrical synapse are largest.

We compared asymmetry, or cc ratios, for all synapse locations as a function of synapse strength (Fig. 2*D*). As expected, connections between the same compartments (e.g., M-M) were perfectly symmetrical for all values of electrical synapse conductance. In contrast, mismatched synapses are marked by decreasing or increasing cc ratios, with the mirror cases producing similar degrees of effective asymmetry in opposite directions (e.g., M-D and D-M). We also noted that asymmetry was greater for more-mismatched synapse pairs (e.g., S-D and M-D, or the blocks in Fig. 2*D*), as the distal-soma connections produce cc ratios furthest from 1. Further, asymmetry was greatest for synapses connected to the soma, while M-D synapses showed a lesser degree of effective asymmetry. These simulations demonstrate that effective asymmetry between somatic integrators can arise from difference in synapse location, when perfectly symmetrical electrical synapses encounter asymmetrical spatial differences between identical somas and dendrites, and thereby dictate effective asymmetry.

**Figure 3.**
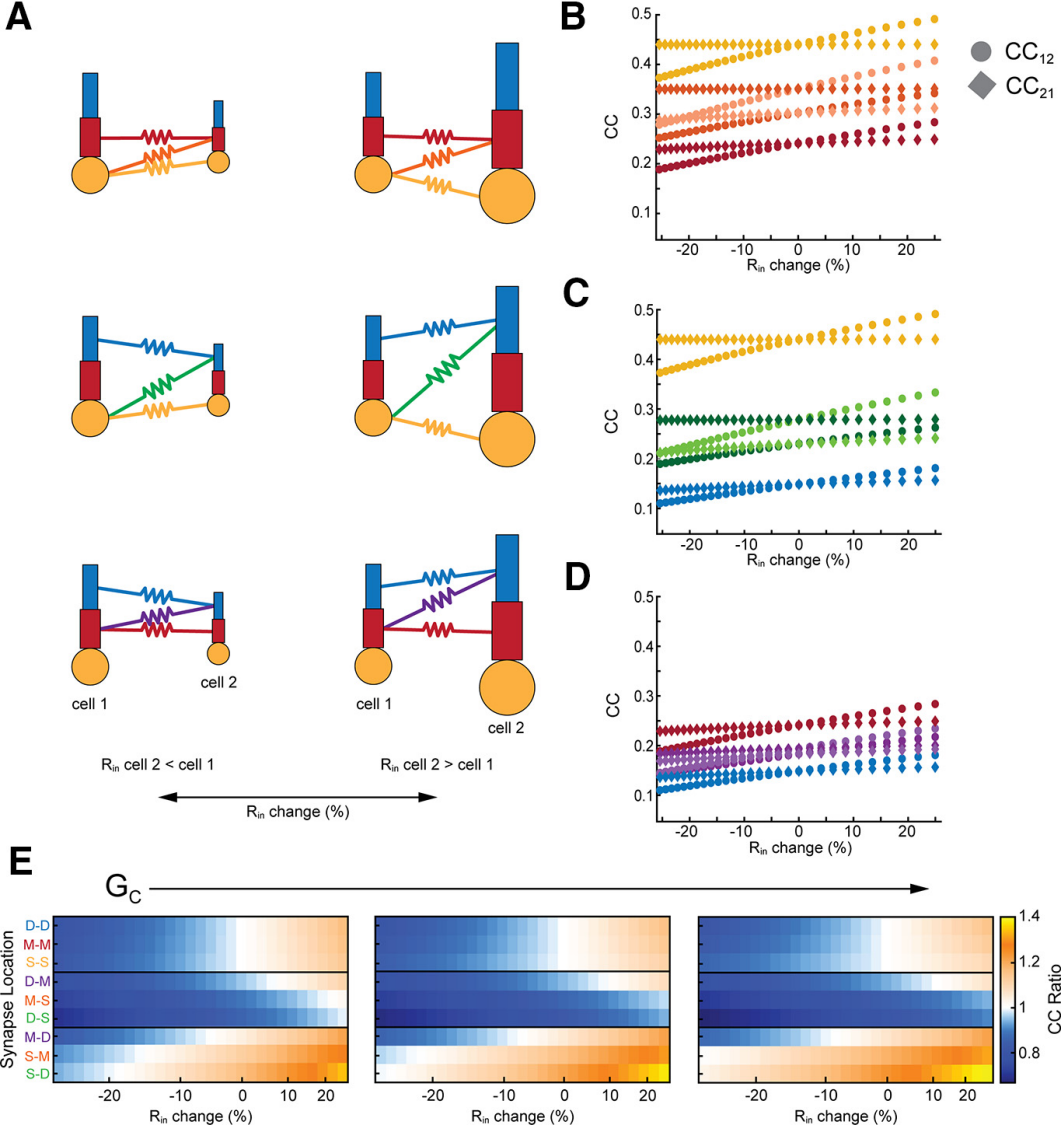
Dependence of asymmetry on synapse location and input resistance differences. ***A***, Schematics depicting differences in input resistance, and varied synapse locations. ***B***, Coupling versus difference in input resistance for GJs (G_C_ = 0.15 mS/cm^2^) between middle-middle, middle-soma, and soma-soma compartments. Difference in input resistance is expressed as cell 2 relative to cell 1. ***C***, As in ***B*** for GJs between distal and soma compartments. ***D***, As in ***B*** for GJs between distal and middle compartments. ***E***, Asymmetry plotted against input resistance differences, grouped by electrical synapse strength (G_C_), which increases across panels: 0.1, 0.15, and 0.2 mS/cm^2^.

Effective asymmetry can also arise from differences in basic excitability, e.g., membrane input resistance R_in_. To demonstrate this widely expected phenomenon, we altered R_in_ by changing leak conductance in cell 2 of the model (Fig. 3*A*), and measuring coupling coefficient cc in both directions. When GJs coupled two somas of differing R_in_, cc was determined only by R_in_ of cell 2 (Fig. 3*B*, yellow); cc_12_ varied, while cc_21_ stayed constant. As GJs were more distant from the soma, voltage divisions allowed both cc_12_ and cc_21_ to change, although changes in cc_12_ were always larger. Differences in GJ location also contributed to asymmetry here, again splitting the differences between the extremes, similarly to the effect shown in Figure 2.

The combined effects of input resistance and location are summarized in Figure 3*E*, which shows simulations for three values of average electrical synapse strength G_C_ for all synapse locations and input resistance mismatches. For matched-compartment locations (top boxes), asymmetry was determined only by differences in R_in_. For GJs that coupled cells with differing Rin and synapses at mismatched locations, synapse location appeared to be a weaker effect than input resistance mismatch: the cell with the GJ closer to its soma always yielded a smaller coupling (e.g., middle box: synapses are closer to soma 2, and produced asymmetry <1). As in Figure 2, asymmetry was strongest for synapses coupling the most spatially separate compartments. These simulations showed us that increasing G_C_ amplified the asymmetry produced by differences in R_in_. Synapses that were mostly below one in cc ratio further decreased in cc ratio (Fig. 3*E*, middle rows), while locations with cc ratio above one increased with the strength of the synapse (Fig. 3*E*, bottom rows). Synapses between similar compartments (top rows) showed minimal changes with increasing strength of the synapse.

**Figure 4.**
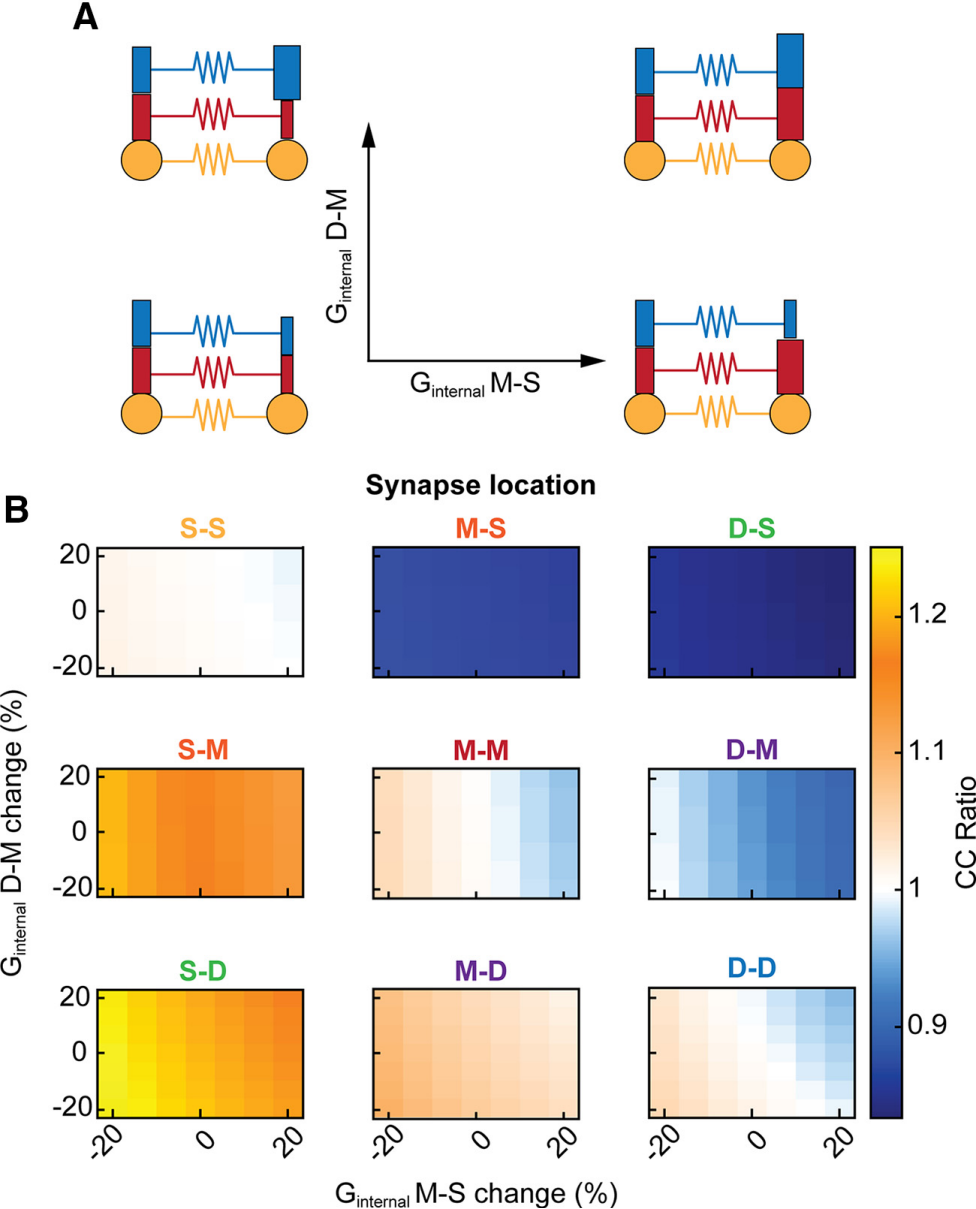
Differences in dendritic morphology fine-tune coupling coefficients and asymmetry. ***A***, Internal conductances between the three subcellular compartments were altered in cell 2, as shown in schematics. Changes in middle-soma conductance is plotted on the *x*-axis, and change in distal-middle conductance is plotted on the *y*-axis. ***B***, Heat maps of cc ratio for all synapse locations. In all cases, decreasing internal conductance increased cc ratio, while increasing internal conductance in decreased cc ratio. G_c_ was 0.15 mS/cm^2^ for all synapses in these simulations.

To further examine how heterogeneity between two coupled cells could contribute to effective asymmetry, we altered the internal coupling conductance between compartments of the cell. For all synapse locations, differences in dendritic coupling altered resulting cc ratios (Fig. 4), but by amounts smaller than synapse mismatch or input resistance difference. Increasing dendritic conductance favors transmission into that cell and thus lowers cc ratio when cell 2 has more-conductive dendrites. Similarly, cc ratio increases when cell 1 is higher in dendritic conductance. This result is consistent for the connections between same cellular compartments, which are symmetric when morphology is the same, and the mismatched locations which are asymmetric in the same case. Although morphology may not produce substantial asymmetry alone, in conjunction with synapse location the intrinsic differences between two cells will fine-tune the overall coupling and asymmetry measured between them.

**Figure 5.**
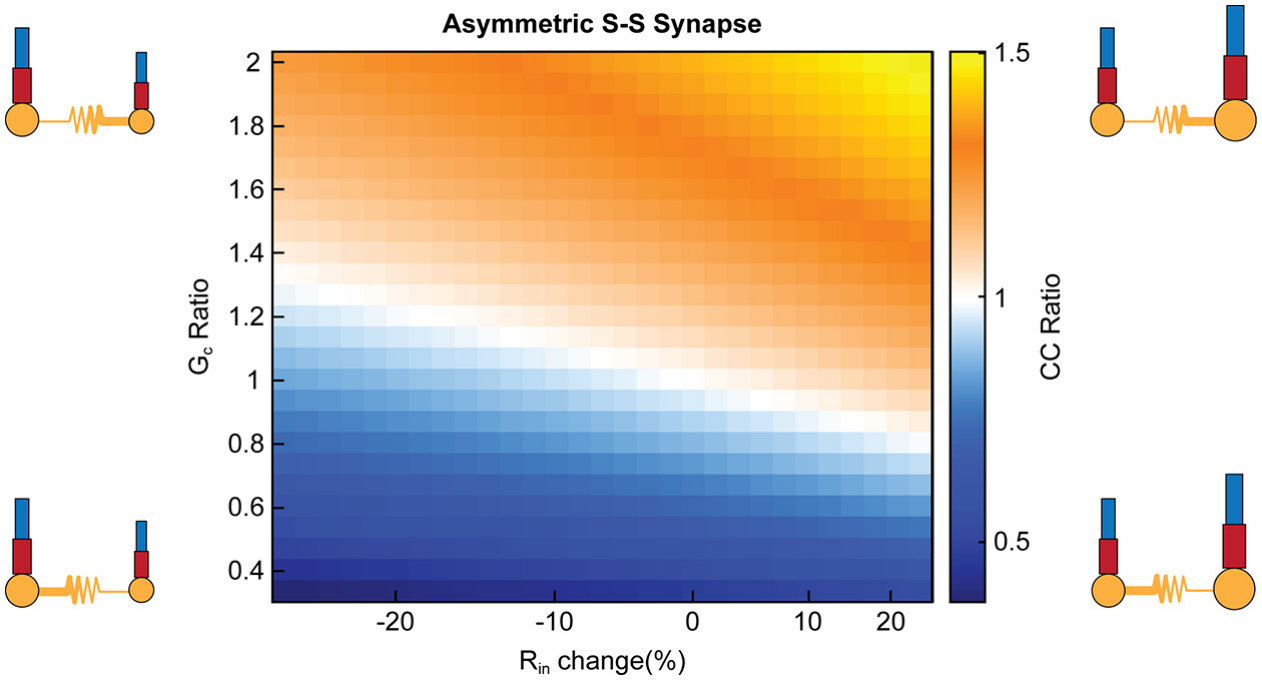
Asymmetry due to altering directional conductance produces similar response in coupling coefficients compared with synapse location asymmetry. Directional conductance changes between the cells produces a similar degree of asymmetry in cc ratio, with input resistance difference between the two cells predictably shifts the cc ratio values. G_c_ ratio (Gc_12_/Gc_21_) from altering conductance from cell 1 to cell 2 was varied, while the opposite direction was held constant (Gc_21_ = 0.15 mS/cm^2^). Input resistance was altered in cell 2 relative to cell 1. Neuron schematics depict direction with larger conductance as a thicker resistor symbol, while size of the cell indicates change in input resistance.

The previous sets of simulations used a symmetrical synapse to show that several aspects of cellular properties and synapse locations can yield effective asymmetry, as expected. Next, we asked whether an electrical synapse that was itself asymmetrical could produce the same effective asymmetries. We varied the conductance G_C_ of the electrical synapse between somatic compartments, and again examined the effect of input resistance changes on effective asymmetry. Our results demonstrate that similar values of effective asymmetry could arise from either G_C_ ratio or input resistance difference (Fig. 5). For each set of input resistances (Fig. 5, column), synaptic asymmetry could produce a range of effective asymmetry. These simulations illustrate the potential for cc values recorded from the soma to appear similarly asymmetric, whether asymmetry is produced from differential intrinsic properties or synapses themselves.

**Figure 6.**
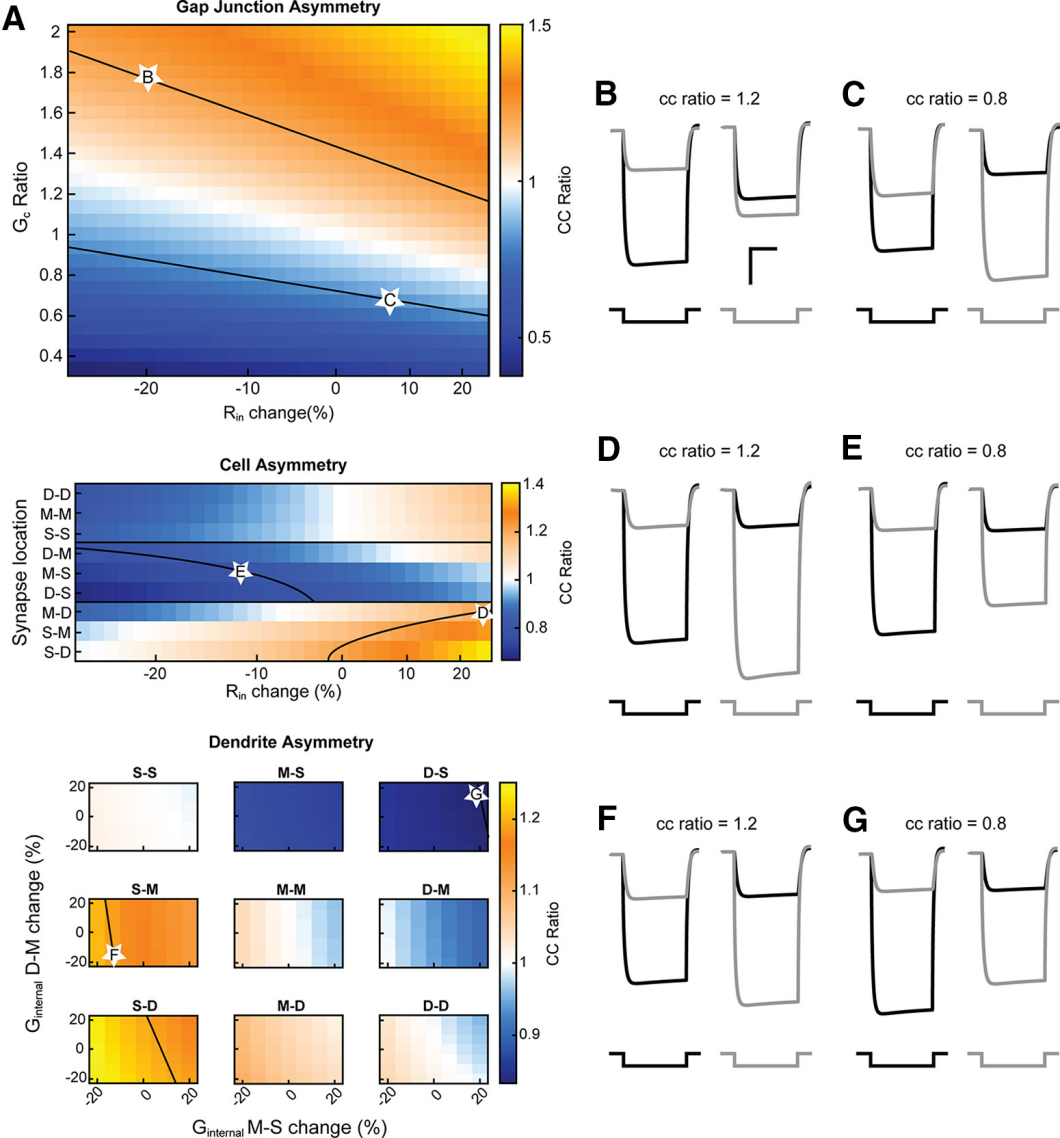
Varied scenarios can produce identical asymmetry as measured at the soma. ***A***, Approximate isoclines (black lines) show parameters that produce the same degree of asymmetry (cc ratio of 1 ± 0.2). Representative traces (***B–G***) were taken from data along these isoclines (white stars) for cases of directionally asymmetrical conducting GJs, differing synapse locations, or differing dendritic geometries. ***B***, G_C_ ratio 1.8, R_in_ change −20%. Scale bars: 5 mV, 200 ms. ***C***, G_C_ ratio 0.667, R_in_ change +6%. ***D***, M-D GJ: R_in_ change +25%. ***E***, M-S GJ, R_in_ change −12%. ***F***, S-M GJ, G_MS_ change −13.3%, G_DM_ change −20%. ***G***, D-S GJ, G_MS_ change +20%, G_DM_ change +20%.

Together, the previous results show that asymmetry in coupling as measured between somas can arise from a number of factors. We demonstrate this masking in Figure 6 the same amount of asymmetry in coupling as measured at the soma can arise from independent sources. We identified simulations that resulted in 20% coupling difference, as this is the most common cc ratio observed in paired recordings at TRN (Haas et al., 2011). Higher transmission to cell 2 by the same proportion (cc ratio ∼1.2) can be produced by asymmetric GJ with G_c_ ratio of 1.8 and R_in_ change of −20% (Fig. 6*B*), or M-D synapse location and +25% R_in_ change (Fig. 6*D*), or S-M synapse with higher dendritic conductance in cell 1 (Fig. 6*F*). Alternatively, higher transmission to cell 1 (cc ratio ∼0.8) can be produced by asymmetric GJ with G_c_ ratio of 0.67 and +6% R_in_ change (Fig. 6*C*), or M-S synapse and −12% R_in_ change (Fig. 6*E*), or D-S synapse with higher dendritic conductance in cell 2 (Fig. 6*G*). Thus, asymmetry measured at the soma is not informative as to its source, and more pertinently, fails to provide insight into processing in coupled dendrites.

**Figure 7.**
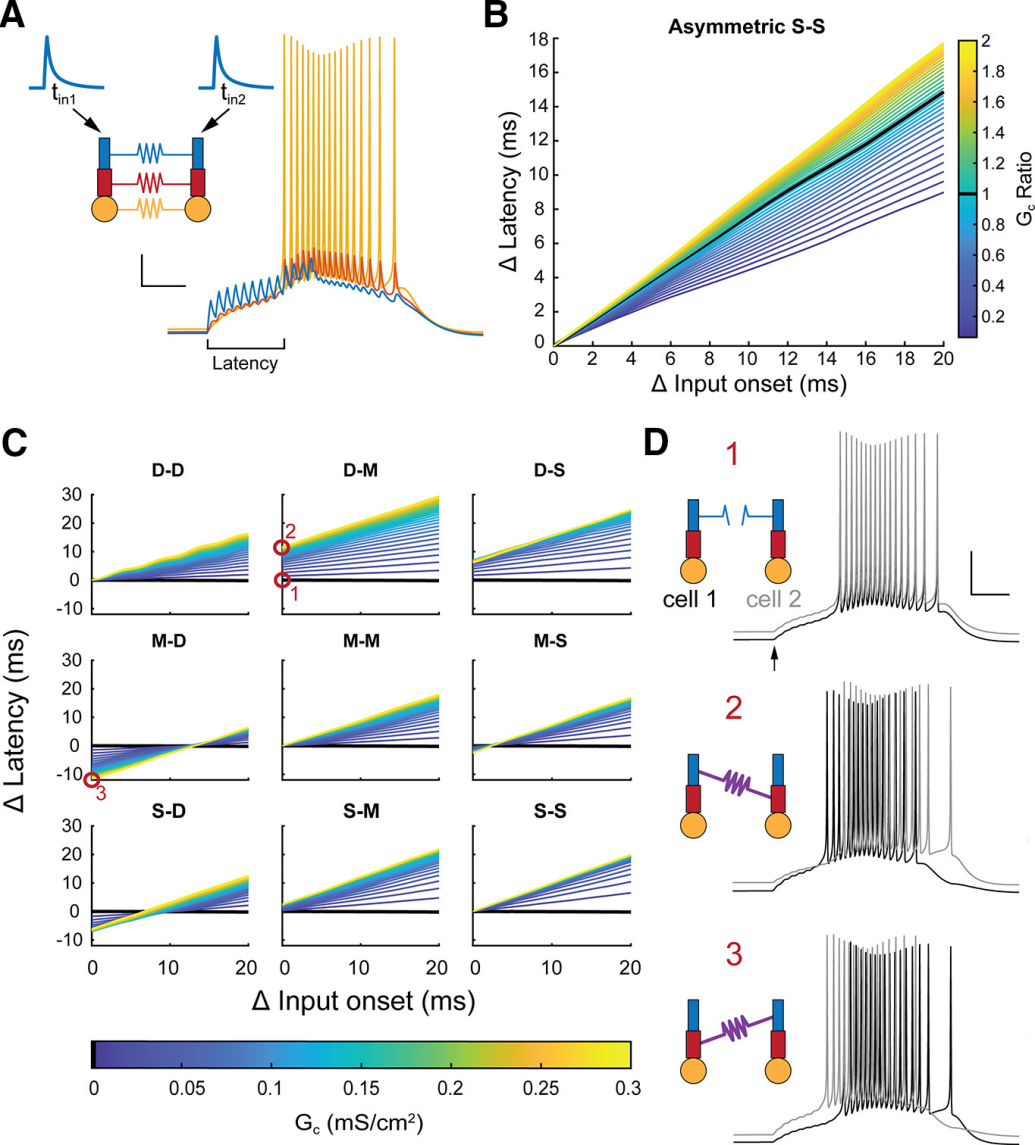
Asymmetry controls the latency modulation produced by electrical synapses. ***A***, Each TRN cell received AMPAergic burst-like inputs (13 events with 1-μA amplitude, 5-ms ISI) at varied onset times. Traces at right show responses of all compartments of a single cell to a burst input. Scale bars: 10 mV, 25 ms. ***B***, Differences in spiking latency between the two cells plotted against difference in onset of burst inputs (t_in2_ – t_in1_) for the two TRN cells. Asymmetrical somatic GJs increased GJ-mediated latency differences for G_C_ ratios >1, and decreased them for G_C_ ratio < 1. ***C***, Changes in latency for varied electrical synapse locations, strengths and input time differences. ***D***, Example bursts taken from ***C*** (red circles) for an uncoupled pair (1), a pair with a high conductance D-M synapse (2), and high-conductance M-D synapse (3). Asymmetrical location of an electrical synapse determines bursting order, and results in positive or negative latency change. Scale bar: 20 mV, 25 ms, traces offset for clarity.

Next, we examined the impact of asymmetry on the function of coupled pairs. Electrical synapses have been previously shown to modulate latency of action potentials in coupled pairs (Haas, 2015; Sevetson and Haas, 2015; Alcami, 2018). We measured latency to burst-like input patterns of AMPAergic synaptic currents delivered to distal dendrites of both cells of a coupled pair, to mimic excitatory afferent activity received by cells of the TRN (Gentet and Ulrich, 2003) from bursting thalamic relay cells (Fig. 7*A*). We tested each synapse location, and also varied the arrival (onset) times of the AMPAergic bursts between the two cells. For synapses between the similar compartments (Fig. 7*C*,*D*), latency difference increases with G_C_ and difference in input time. Dissimilar locations alter the latency modulation with a variety of effects, with synapses in varied locations shifting latency modulation curves either up (D-M) or down (M-D), producing a variety of outcomes in cell firing by as much as 20 ms. This trend is generalized in the asymmetrically conducting synapse, where G_C_ ratio >1 produces higher latency modulation, and values <1 shift latency modulation lower (Fig. 7*B*).

**Figure 8.**
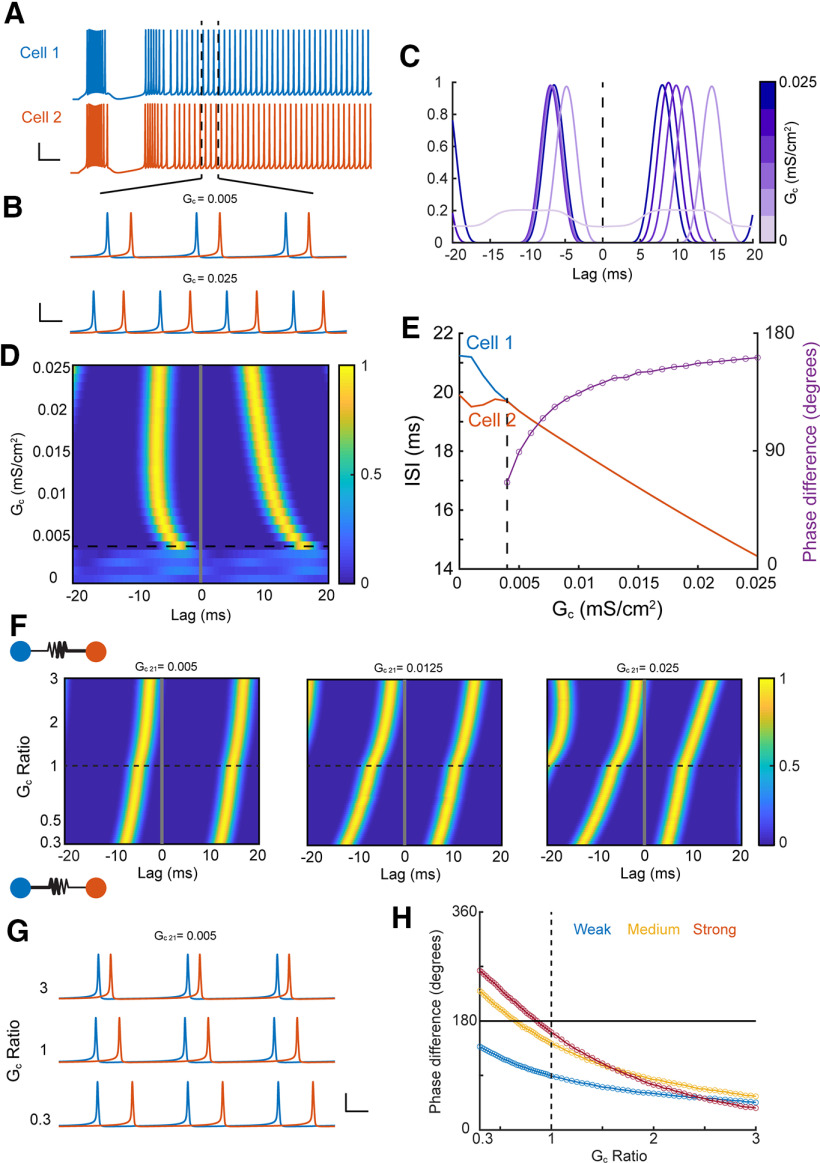
Asymmetry determines the phase of synchrony. ***A***, Traces of two coupled TRN cells driven to spike tonically; cell 2 received a slightly larger current pulse. I_1_ = 0.575, I_2_ = 0.6 μA/cm^2^. Scale bars: 25 mV, 50 ms. ***B***, Zoom of tonic spikes from ***A*** showing synchronous firing for low coupling strength (G_c_ = 0.005 mS/cm^2^) and out-of-phase synchrony for higher coupling strength (G_c_ = 0.025 mS/cm^2^). Scale bars: 40 mV, 5 ms. ***C***, Cross correlation of tonic spikes for varied values of symmetric GJ strength. ***D***, Histogram of cross correlations for a symmetric synapse. ***E***, ISIs for tonic spikes and phase difference (purple) of synchronous spike trains, dotted line indicates the emergence of synchrony from cross correlations in ***D***. ***F***, Cross correlations for asymmetric synapses. Asymmetry values >1 represent better transmission from cell 1 to cell 2. Initial strength of the GJ, G_c 21_, increases across panels. ***G***, Traces from weakly coupled cells in ***F*** for three different asymmetry conditions. Scale bars: 40 mV, 5 ms. ***H***, Phase difference for asymmetric GJs from ***F***, for weak (blue, G_c 21_ = 0.005), medium (yellow, G_c 21_ = 0.0125), and strong (red, G_c 21_ = 0.025) initial synapse strengths.

To examine the possible consequences of asymmetry on spike synchrony, a well noted function of electrical synapses, we used single-compartment models used previously (Haas and Landisman, 2012; Pham and Haas, 2018) to analyze correlations of tonic spike trains (Fig. 8*A*,*B*) elicited by steady current injection, with one cell (here, cell 2) driven slightly faster. Synchrony was demonstrated by peaks in steady-state cross-correlation of the spike trains (Fig. 8*C*,*D*). As electrical synapse strength increased, spike rates of the two neurons converged for coupling strength larger than 0.004 mS/cm^2^, and increased together with synapse strength because of the increase in excitability contributed by the GJ (Fig. 8*E*). As expected from theoretical models of coupled oscillators (Lewis and Rinzel, 2003; Saraga and Skinner, 2004), our simulations revealed synchronous firing that transitioned from stable in-phase (∼0° lag) to out-of-phase (∼180° lag) forms (Fig. 8*E*) for a symmetrical GJ. We next observed that asymmetry of the GJ interacted with strength and altered the form of synchrony (Fig. 8*F*). For weak coupling, asymmetry that increased from 0.3 to 3, altering the identity of the favored cell, brought spike times closer together and produced a transition from out-of-phase to in-phase synchrony. The impact of asymmetry strengthened as synapse strength increased (Fig. 8*F*, across panels). Asymmetry that favored transmission arising from the slower cell (G_C_ ratio > 1) brought firing closer to in-phase, while asymmetry that favored the faster cell (G_C_ ratio < 1) led to out-of-phase solutions (Fig. 8*G*,*H*). These results together show that asymmetry, regardless of its subcellular source, controls synchronous rhythmic activity between coupled neurons.

## Discussion

Asymmetry of transmission at electrical synapses has been widely noted but its specific sources rarely explored in depth, perhaps because of the experimental difficulties of identifying and localizing specific GJs *in vitro* or *in vivo*. Nonetheless, because asymmetry is pervasive and can result in extreme cases in which spikes in one cell more or less faithfully drives spiking in the coupled neighbor (Apostolides and Trussell, 2013; Rash et al., 2013), we sought to understand how basic neuronal properties could influence effective coupling, and thereby the function of coupled networks. Here, we have shown that asymmetry can arise from a variety of intrinsic differences in neuronal properties as well as differences in subcellular localization of the GJ between somas. We expect additional heterogeneities, such as in the ionic currents expressed in each cell, will similarly affect coupling measurements and thus effective asymmetry, as similar activity patterns can be produced by a variety of models in pyloric circuit (Prinz et al., 2004). In practice, asymmetry is a combined product of all of these factors together. We also found that asymmetrical and/or strong synapses between dendritic compartments can be masked from somatic detection by the same intrinsic properties. Our measurements here focused on soma-to-soma transmission, as ultimately, asymmetry between somas is the last stop before spike generation in the axon initial segment, and because electrical synapse strength is traditionally measured between somas. Indeed, our results also highlight that regardless of its source, asymmetry substantially impacts spike times and synchrony between coupled cells (Gutierrez and Marder, 2013; Sevetson and Haas, 2015).

Precise locations of electrical synapses along dendrites have proven difficult to exhaustively determine, but a handful of studies point toward asymmetrical localization. In coupled interneurons of cortex Layer IV, synapses are located all along the dendrites, and measurements from 204 cells showed strong asymmetry in localization, with 90% synapses within 50–75 μm of one soma, but up to 250 μm away from the coupled soma (Fukuda, 2017). Asymmetrical localization also appears to be a feature of coupling between cerebellar Golgi cells (Szoboszlay et al., 2016). The strongly asymmetrical synapses of the DCN also appear to couple mismatched distances from fusiform and stellate somas (Apostolides and Trussell, 2013). Other studies indicate that dendritic location of GJs is diverse across brain areas, and thus asymmetry could vary widely. In brainstem MesV cells, GJs appear to be located at or very close to the soma (Curti et al., 2012). In contrast, in inferior olive (Devor and Yarom, 2002; Hoge et al., 2011) cells are coupled at quite distal dendrites, such that somatic measurements of coupling themselves are small. Average intersomatic distances between coupled cells in TRN are ∼100 μm (Lee et al., 2014), implying that GJs are dendro-dendritic, and have a great deal of potential to create asymmetric localization of GJs between cells.

Dendritic integration is likely to be influenced by the presence of GJs along dendrites (Vervaeke et al., 2012), as they have been shown to act as a shunt of current arising from nearby chemical synapses (Llinas et al., 1974; Lang et al., 1996), and in *C. elegans* coupled motor neurons, electrical synapses spread excitation during contraction and inhibit cell pairs between cycles through a shunting effect (Choi et al., 2021). Asymmetry has also been shown to amplify EPSPs in mixed synapses (Liu et al., 2017). Additionally, dendritic morphology determines transmission across GJs (Nadim and Golowasch, 2006), as well as firing patterns of extended morphologic models (Mainen and Sejnowski, 1996), and as our results demonstrate, substantial coupling influences on dendritic processing may not be appreciably indicated by somatic measurements.

Effective asymmetry results in differentially directed signal and information flow through a network that includes realistic electrical coupling. Our results here raise interesting questions whether cells within a network regulate any of the factors that result in asymmetry to produce precise direction of information flow within their network. Increasing electrical synapse strength through trafficking of connexin proteins, a process which is controlled by cAMP expression (Palumbos et al., 2021), may determine location or possibly effectively relocate a synapse slightly closer to or distal from the soma. Distances of dendritically located electrical synapses between cerebellar Golgi cells do not correlate with coupling strength measured between somas (Szoboszlay et al., 2016), indicating a possible compensation for distance by strength upregulation for those cells. Further, our previous work demonstrating activity-dependent plasticity showed that asymmetry changes systematically with unidirectional activity or ion flow across the GJ (Haas et al., 2011; Fricker et al., 2021). Those results imply that asymmetry is a modifiable element of electrical synapse plasticity. Our results here also point out that cellular changes, such as activity-induced changes in dendritic resistance or mutation-induced localization of GJs, could result in the changes in asymmetry measured, in addition to the possibility of changing the conductance itself.

Asymmetry, as it influences spike times in coupled cells, has downstream effects on the synaptic targets of the coupled cells. Symmetrical electrical synapses between model TRN cells act to merge spike times of thalamocortical cells in response to inputs of similar strength or timing, or can separate spikes from dissimilar inputs (Pham and Haas, 2018). We hypothesize that TRN neurons with asymmetric GJs will inhibit thalamocortical relay cells unequally, shifting the balance between merging or distinguishing signals as they are relayed to cortex. Including asymmetry as a factor in TRN networks will be important to understanding how TRN cells orchestrate the attentional spotlight at sensory thalamic nuclei. In canonical feedforward circuits, coupling between inhibitory interneurons impacts integration in principal cells (Pham and Haas, 2019). Recent investigations further show the influence of electrical synapses on temporally precise inhibition in feedforward circuits (Hoehne et al., 2020; Chagnaud et al., 2021). Asymmetry, as it can be applied to electrical synapses in these general motifs, may impact the many GJ coupled feedforward and feedback circuits that embed electrical synapses across the brain.

## Synthesis

Reviewing Editor: Arianna Maffei, Stony Brook University Decisions are customarily a result of the Reviewing Editor and the peer reviewers coming together and discussing their recommendations until a consensus is reached. When revisions are invited, a fact-based synthesis statement explaining their decision and outlining what is needed to prepare a revision will be listed below. The following reviewer(s) agreed to reveal their identity: Farzan Nadim.

The reviewers agreed that this is a compelling study reporting a model for a systematic examination of the properties of electrical synapses. They raised a series of concerns that require attention. In particular, one of the reviewers found that little emphasis was given to what they identified as interesting and novel results reported in some of the figures.

Detailed feedback by the reviewers is provided below.

Reviewer #1:

The article ’Intrinsic sources and functional impacts of asymmetry at electrical synapses’ presents a systematic examination of several factors that result in, or counteract, functional asymmetry at electrical synapses, and explores some functional consequences of asymmetry. To this end, the authors use a simplified multi-compartmental model of thalamic reticular nucleus neurons.

The article has the merit to provide a systematic evaluation of electrical synapse asymmetry and is a valuable contribution to the electrical synapse field. A number of results presented in the article are based on well-established theoretical predictions that have been partly described in early and more recent work. These results are expected and present relatively little novelty. Later sections/figures build up from previous ones and describe interesting results, exploring the parameter space that influences asymmetry and its consequences.

I have three concerns, which should in my opinion be addressed before publication:

1) The article overemphasizes in different places some of the results, making claims that read like overstatements, and does not always acknowledge previous work as detailed below. The authors may want to emphasize the novelties found in the last figures. There is in my opinion too much emphasis on aspects that are not as new as the authors claim (nor accurately referenced at times), mainly the first figures, and not enough emphasis for the novelty of the findings shown in the last figures.

2) It should be acknowledged that the model is simple and not morphologically-detailed. Moreover, some parameters should be explicitly provided in the text and compared to real values to justify that this simplification captures the properties of coupled TRN neurons.

How did the input resistance compare with previously experimentally-measured values and how justified are the access resistance values to the dendritic compartments and their cable properties? Are they in agreement with electrophysiological measurements and reconstructed TRN cells dendrite diameters and lengths? Both input and access resistances to the compartments are critical in modulating the magnitude of effects explored along the paper and should be correctly justified with anatomical/physiological values from the literature. Mismatch of real neurons and the model for these parameters may produce unrealistic results. Amsalem et al. (2016) estimate single-cell intrinsic input resistance of coupled cells embedded in a network in order to model accurately single cells with the appropriate excitability in a network model. Was this considered here somehow, and did the effective input resistance match measured ones, with realistic values of coupling resistance and number of coupled cells?

Related to the previous point, it seems that the authors used a 2-cell model from which they characterize asymmetry, instead of two cells embedded in a a network model of the TRN connected network (number of connections, size of network). If this is the case, the model is likely to include errors in modeled cells and in the phenomena characterized in the article. Results and their magnitude should be confirmed in a realistic network model in terms of connected neighbors and conductance.

Why was the membrane capacitance adjusted to 1.2 μF/cm2 and not 1μF/cm2?

3) Finally, I think that the term synchrony is not correctly used in the presentation and interpretation of results from Figure 8, the section/figure should be rewritten accordingly.

Additional comments:

1) (line 18) What do the authors mean with last sentence of the abstract? How should the results be considered to modify experimental demonstrations of coupling? Same applies for ’when assembling networks containing electrical synapses’.

2) (line 45) Dugué et al. do not report asymmetrical coupling to my knowledge; Szoboszlay et al. report a relatively small coupling asymmetry in coupling coefficients (∼1.3). In the cerebellum, molecular layer interneurons show stronger asymmetry and could be also quoted (ratio of ∼3.3 in half of the pairs in Mann Metzer and Yarom, 1999 where ratios below 1.25 had been considered symmetric; ratio ∼1.7 in Alcami and Marty, 2013). I think that M. Bennett in earlier work already noted that difference input resistances would lead to asymmetry.

3) (line 74) ’In all, asymmetry and its sources remain underexamined’ is not really correct, nor consistent with the paragraph. Measurements of asymmetry, and possible sources of asymmetry have been provided in a number of articles, many of which are acknowledged by the authors themselves: i) relation with input resistances: e.g. Veruki and Hartveit, Mann-Metzer and Yarom, which I suggest to be cited for this, etc; ii) asymmetric modulation of connexin properties on both sides of the gap junction: Palacios-Prado et al.; iii) a possible technical source of error in asymmetry measurements not mentioned in the article has been suggested by Alcami and Marty: an asymmetry of leak conductance.

4) (line 96, line 219) change in temporal precision/latency by electrical synapses was also shown in the cerebellar basket network by Alcami (2018).

5) (abstract, line 104) What do authors mean by ’true’ asymmetries? The asymmetries of conduction across the gap junction? All asymmetries are ’true’, but defined differently.

6) (line 105-106) ’Together, these results underline that one should in fact always expect asymmetry at almost any electrical synapse’ is a strong statement: depending on the values of biological parameters, in some cases this asymmetry can be small and no significant asymmetry expected. I suggest to rephrase, if this is what authors meant, maybe by ’Together, these results underline that effective asymmetric transmission, defined as the effective asymmetry of transmission between two compartments from coupled cells, is likely to occur in more cases that previously considered.’

7) Legend Figure 1. Replace top and bottom by C1, C2, D1, D2.

8) (line 134) why ’might (one) expect to produce the same coupling’? Doesn’t theory predict the opposite? First figures are predicted by theory, and authors explore them numerically in their model, which is fine. Since some of these effects have been already suggested, theoretically predicted or shown in other networks, they should be presented accordingly.

9) Authors use ’observed’, ’effective’ or ’apparent’ asymmetry. It would be useful to use a consistent word in the manuscript/clarify that the three refer to the same.

10) Legend Figure 3, typo: ’input electrical synapses resistance’.

11) Figure 3, bigger cells to schematize larger input resistances is confusing. Is cell 1 on the left and cell 2 on the right? Since changes performed by the authors only affect leak intrinsic resistance according to the text, authors may want to represent a smaller number of channels on the membrane of the cell with higher membrane resistance, without changing cell size, or represent the leak resistance with different size as they do for the junctional resistance.

12) Do 8A,B show synchronous or antisynchronous firing? Coupled cells fire in antisynchrony. C displays no measure of synchrony. Similar ISIs do not mean synchrony. Moreover, how do the authors decide who is leading? If I understand well, both cells lead for the authors, depending on the perspective, but then there is stricto sensu no leading cell, since both lead and follow. Does the term make sense to determine firing order in a tonic train, and with antisynchronous spikes? This should be clarified and corrected, related synchrony statements are in my view misleading. In fact, panel E suggests transition from antisynchrony to synchrony as one cell is made a clear leader by increasing synapse asymmetry. Could authors generate a plot to visualize synchrony?

13) Figure 8 legend: there is a typo: ’from cell , the initially- cell, to cell .’ Cell IDs are missing.

14) (line 271-275) Reading these lines, it seems that asymmetry is not the general case in networks that authors quote, but authors deduce that ’asymmetrical location is a feature of coupling’.

15) (line 281) ’Dendritic integration is likely to be influenced by the presence of gap junctions along dendrites’ --this has been shown, e.g. by Vervaeke et al. (2012). The article should be quoted and added to the references.

16) (line 289) idem, previous literature demonstrating this should be quoted.

17) (line 291) ’Our results...’ is confusing and should be rewritten.

Reviewer #2

In this computational modeling study, the authors address the potential sources of the experimental observation of asymmetric electrical synapses and its consequences for circuit activity. Towards this, the authors first consider biologically plausible sources of asymmetry, focusing on intrinsic (morphology, compartment structure, and membrane resistance) and synaptic properties (coupling strength and location). Potential functional consequences are addressed, focusing on how spiking behavior can be altered by asymmetric electrical synapses.

The authors employed biophysical models and found that several distinct mechanisms can potentially give rise to asymmetric electrical synapses, which underscores the experimental challenge of pinpointing the precise biological mechanisms at play. This challenge is further highlighted by their demonstration of the difficulty of recovering a ground-truth asymmetric electrical synapse in even the simplest of scenarios (Figure 5). The authors go on to show that asymmetric electrical synapses can modify the spike latency and order of coupled bursters, which is extended to demonstrate the influence of asymmetric electrical synapses in the case of synchrony of tonic spikers.

This is a well written manuscript that systematically addresses the degeneracy of asymmetric electrical synapses in a way that would be experimentally infeasible. It provides the field with a framework for better understanding how electrical synapses contribute to neural circuit function. We have only minor issues that are listed below.

Minor comments:

The simulations were mostly done with models of the same intrinsic properties except where explicitly specified. This is completely fine for the sake of demonstrating the points posed in the manuscript. However, it would be useful to the field to briefly discuss how these results would be influenced by heterogeneity in the intrinsic properties (ex. I_k, I_Ca, ... ) of the coupled cells.

Line 32: The first sentence of the introduction is very “mammalian” oriented, but the results clearly extend beyond GABAergic cells

Line 34: Rectification is mentioned, but what is being considered here is not necessarily classical rectification as in a diode and as in the literature cited. In this manuscript, the asymmetry is modeled by unequal strength of gap junction conductance, and not rectification where current flows preferentially in a given direction.

Line 38 stomatogastric ganglion instead of nucleus

Line 84: missing a period after the Mamiya reference

Line 99 or better Line 113 I missed a bit more info about the model (spiking/non spiking, LIF?) short description of properties, at least which currents are included.

Fig 1C which cell is cell1, not labeled. why headline D-D Synapse over voltage in C1 and C2 (also not specified in figure legend), as far as I understand it the current injection in C1 is into the soma and the colors represent the synapse location?. + it took me a while to figure out that the left voltage traces are cell 1 (I guess) and the right voltage traces are cell 2.

May be worth including some equations in-line for clarity (ex. how asymmetry was modeled)

Line 145/153 Is asymmetry defined by cc ratios? Line 152/153 talks about D-M synapses showed “lower values of asymmetry” meaning the cc ratio is closer to 1 than s-d. I have a problem with the term “lower values” for the cc ratios >1, (which is a bigger value in cc-ratio speak, but not in their use of “asymmetry”).

Fig 2/3 their labeling some-middle synapse etc isn’t correct as they also show s-s and m-m etc. they are only limiting the possible synapse location to two of the three possibilities.

Fig 2 the two shades of orange/green are hard to distinguish.

Line 217 ff/fig 7 again having troubling understanding what was done. Are both cells receiving bursting input? What does differing onset times mean? Fig 7a shows one set of voltage traces -> cell 1 or 2? Did they give the same bursting input in cell 1 and (?) in cell 2 time shifted and measured when the soma started spiking (which?)?

Fig 7D colors confusing as yellow and blue used in other contexts. Greyshades + neuron & synapse schematic would be helpful (instead of cell1, cell2 and connection only mentioned in figure legend). Figure legend refers to top, middle and bottom, figures labeled with 1, 2, 3.

Line 308 what’s TC spike times?

Line 224 refers to fig 7b, D-D, there’s no D-D in B,

Line 325ff very hard to figure out which value in line 327 and 328 belongs to currents listed in 325 ff

Line 331 units are missing

## Author Response

Synthesis of Reviews:

Computational Neuroscience Model Code Accessibility Comments for Author (Required):

The authors indicate that the Model Code is made accessible. However, both reviewers were not able to access it.

The code should be available now in its final form on our lab’s GitHub repo.

Significance Statement Comments for Author (Required):

The reviewers found that the study is very useful as it elucidates the sources of asymmetry when measuring electrical coupling through gap junctions. It was recognized as “quite handy in making experimentalists aware of these different possibilities”.

Comments on the Visual Abstract for Author (Required):

N/A

Synthesis Statement for Author (Required):

The reviewers agreed that this is a compelling study reporting a model for a systematic examination of the properties of electrical synapses. They raised a series of concerns that require attention. In particular, one of the reviewers found that little emphasis was given to what they identified as interesting and novel results reported in some of the figures.

Detailed feedback by the reviewers is provided below.

Thank you for the thorough evaluation, and for the feedback that has helped to improve our work. Our responses are in blue text below.

Reviewer #1:

The article ’Intrinsic sources and functional impacts of asymmetry at electrical synapses’ presents a systematic examination of several factors that result in, or counteract, functional asymmetry at electrical synapses, and explores some functional consequences of asymmetry. To this end, the authors use a simplified multi-compartmental model of thalamic reticular nucleus neurons.

The article has the merit to provide a systematic evaluation of electrical synapse asymmetry and is a valuable contribution to the electrical synapse field. A number of results presented in the article are based on well-established theoretical predictions that have been partly described in early and more recent work. These results are expected and present relatively little novelty. Later sections/figures build up from previous ones and describe interesting results, exploring the parameter space that influences asymmetry and its consequences.

Thank you for the thorough evaluation of our work.

I have three concerns, which should in my opinion be addressed before publication:

1) The article overemphasizes in different places some of the results, making claims that read like overstatements, and does not always acknowledge previous work as detailed below. The authors may want to emphasize the novelties found in the last figures. There is in my opinion too much emphasis on aspects that are not as new as the authors claim (nor accurately referenced at times), mainly the first figures, and not enough emphasis for the novelty of the findings shown in the last figures.

Thank you for your comment, and for aptly noting this transition in the paper. We have added text to more clearly indicate the transition from our elaboration of expected effects, to the novel findings later. In the introduction, we have used (line 103) “as predicted", “We then show” (104-5) and “unexpectedly” (line 110) to better mark the contrast. In the Results, we added “To demonstrate this widely expected phenomenon” to the section on varied Rin, and “as expected” where we transition into showing compensation (Fig. 5). We hope this is sufficient to mark the distinctions between our less-novel and more-novel results.

2) It should be acknowledged that the model is simple and not morphologically-detailed. Moreover, some parameters should be explicitly provided in the text and compared to real values to justify that this simplification captures the properties of coupled TRN neurons.

How did the input resistance compare with previously experimentally-measured values and how justified are the access resistance values to the dendritic compartments and their cable properties? Are they in agreement with electrophysiological measurements and reconstructed TRN cells dendrite diameters and lengths? Both input and access resistances to the compartments are critical in modulating the magnitude of effects explored along the paper and should be correctly justified with anatomical/physiological values from the literature. Mismatch of real neurons and the model for these parameters may produce unrealistic results. Amsalem et al. (2016) estimate single-cell intrinsic input resistance of coupled cells embedded in a network in order to model accurately single cells with the appropriate excitability in a network model. Was this considered here somehow, and did the effective input resistance match measured ones, with realistic values of coupling resistance and number of coupled cells?

Related to the previous point, it seems that the authors used a 2-cell model from which they characterize asymmetry, instead of two cells embedded in a a network model of the TRN connected network (number of connections, size of network). If this is the case, the model is likely to include errors in modeled cells and in the phenomena characterized in the article. Results and their magnitude should be confirmed in a realistic network model in terms of connected neighbors and conductance.

Why was the membrane capacitance adjusted to 1.2 μF/cm2 and not 1μF/cm2?

Thank you for these inquiries. We agree that proper model construction is important. We added text to enhance our short description of the model used, found in the first pp. of Results, so that our model type and extent is clear to the reader. We also added text to indicate that our dendritic access and conductance choices were made to match functional responses of the model (backpropogated signals, PSPs, f/I curves) to real TRN dendritic recordings (Connelly et al 2017, and our lab’s own years of recordings), and that coupling matches the range measured in vitro. These are what we considered the most crucial choices to understand how coupling and integration is altered by dendrites. We also increased capacitance, as you mentioned, in the compartmental model in order to obtain longer input-to-spike latencies to work within, as many models only produce spikes at very short latencies that are less physiological. Increasing capacitance does not affect coupling measurements.

Input resistance itself is not an exact match - our model has single-digit values / cm2 while TRN cells are in tens and hundreds of MOhm - and so we tailored our input amplitudes to appropriate peri-threshold levels, which can be seen and evaluated in our figures, in order to evaluate the effects of asymmetry. Together, these give us confidence that our modeled effects are sufficiently realistic.

We appreciate that embedding the present 2-cell work in a larger network model would be ideal and we are indeed extending our work to that level, but it is beyond the scope of the current work. We hope that these 2-cell model results as they are will form a reasonable basis for that work.

3) Finally, I think that the term synchrony is not correctly used in the presentation and interpretation of results from Figure 8, the section/figure should be rewritten accordingly.

We have revised these sections (please see text, starting at line 234), along with the suggestions from your minor comment #12 to expand Figure 8 and its legend. We agree that we needed to provide better definitions of in-phase synchrony and antisynchrony (out-of-phase synchrony), and that using the term ‘leading’ in the context of antisynchrony was not accurate; we have removed it.

Additional comments:

1) (line 18) What do the authors mean with last sentence of the abstract? How should the results be considered to modify experimental demonstrations of coupling? Same applies for ‘when assembling networks containing electrical synapses’.

We appreciate the opportunity to clarify. We have revised the abstract to more clearly indicate that asymmetry should be measured and reported from experiments, and included in computational work: Our findings highlight aspects of electrical synapses that should always be included in experimental demonstrations of coupling, and when assembling simulated networks containing electrical synapses.

2) (line 45) Dugué et al. do not report asymmetrical coupling to my knowledge; Szoboszlay et al. report a relatively small coupling asymmetry in coupling coefficients (∼1.3). In the cerebellum, molecular layer interneurons show stronger asymmetry and could be also quoted (ratio of ∼3.3 in half of the pairs in Mann Metzer and Yarom, 1999 where ratios below 1.25 had been considered symmetric; ratio ∼1.7 in Alcami and Marty, 2013). I think that M. Bennett in earlier work already noted that difference input resistances would lead to asymmetry.

Thank you for this comment. Dugue et al. do describe their results as symmetric coupling, and so that citation has been removed. We have added citations to the suggested studies mentioned here.

3) (line 74) ‘In all, asymmetry and its sources remain underexamined’ is not really correct, nor consistent with the paragraph. Measurements of asymmetry, and possible sources of asymmetry have been provided in a number of articles, many of which are acknowledged by the authors themselves: i) relation with input resistances: e.g. Veruki and Hartveit, Mann-Metzer and Yarom, which I suggest to be cited for this, etc; ii) asymmetric modulation of connexin properties on both sides of the gap junction: Palacios-Prado et al.; iii) a possible technical source of error in asymmetry measurements not mentioned in the article has been suggested by Alcami and Marty: an asymmetry of leak conductance.

We have added these citations, and modified the text to use the term “underappreciated”.

4) (line 96, line 219) change in temporal precision/latency by electrical synapses was also shown in the cerebellar basket network by Alcami (2018).

Thanks, we have added this citation.

5) (abstract, line 104) What do authors mean by ‘true’ asymmetries? The asymmetries of conduction across the gap junction? All asymmetries are ‘true’, but defined differently.

We agree that this was misworded. We removed the word ‘true’, and added the term ‘conductance’.

6) (line 105-106) ’Together, these results underline that one should in fact always expect asymmetry at almost any electrical synapse’ is a strong statement: depending on the values of biological parameters, in some cases this asymmetry can be small and no significant asymmetry expected. I suggest to rephrase, if this is what authors meant, maybe by ’Together, these results underline that effective asymmetric transmission, defined as the effective asymmetry of transmission between two compartments from coupled cells, is likely to occur in more cases that previously considered.’

Thank you, we have rewritten this sentence (now line 108).

7) Legend Figure 1. Replace top and bottom by C1, C2, D1, D2.

Thanks, we have made this substitution.

8) (line 134) why ‘might (one) expect to produce the same coupling’? Doesn’t theory predict the opposite? First figures are predicted by theory, and authors explore them numerically in their model, which is fine. Since some of these effects have been already suggested, theoretically predicted or shown in other networks, they should be presented accordingly.

Here we were referring to an electrical synapse connecting different compartments. Since the cells are identical in input resistance and the electrical synapse is symmetrical in conductance, coupling might be expected to be identical in both directions. As we show, this is not the case, as the dendrite leaks current from the GJ resulting in different measurements of coupling at the soma.

9) Authors use ‘observed’, ’effective’ or ’apparent’ asymmetry. It would be useful to use a consistent word in the manuscript/clarify that the three refer to the same.

Thank you for pointing this out. Several uses of ‘observed’ and ’apparent’ referencing asymmetry have been changed to ’effective’ for consistency.

10) Legend Figure 3, typo: ’input electrical synapses resistance’.

Thanks, we have made this correction.

11) Figure 3, bigger cells to schematize larger input resistances is confusing. Is cell 1 on the left and cell 2 on the right? Since changes performed by the authors only affect leak intrinsic resistance according to the text, authors may want to represent a smaller number of channels on the membrane of the cell with higher membrane resistance, without changing cell size, or represent the leak resistance with different size as they do for the junctional resistance.

We agree that these schematics could be clearer. We have opted to use text to indicate the identity of the cells, and to indicate differences in input resistance. We hope the revised figure is clearer to the reader in identifying the varied parameters.

12) Do 8A,B show synchronous or antisynchronous firing? Coupled cells fire in antisynchrony. C displays no measure of synchrony. Similar ISIs do not mean synchrony. Moreover, how do the authors decide who is leading? If I understand well, both cells lead for the authors, depending on the perspective, but then there is stricto sensu no leading cell, since both lead and follow. Does the term make sense to determine firing order in a tonic train, and with antisynchronous spikes? This should be clarified and corrected, related synchrony statements are in my view misleading. In fact, panel E suggests transition from antisynchrony to synchrony as one cell is made a clear leader by increasing synapse asymmetry. Could authors generate a plot to visualize synchrony?

Thank you for this comment, which has helped us clarify the work. To demonstrate synchrony, we added examples of spike trains and cross correlations to Fig. 8. We have removed the term “leading” from the text (it was mis-leading, as you note). We have rewritten the corresponding text and figure legend to be clear on our definitions and use of the term synchrony - we now distinguish by in-phase and out-of-phase synchrony.

Please note that the heat maps in Figure 8 appear higher-contrast than in our previous submission. We realized that we had taken cross correlations over only one interval of spiking in the previous version, and now use several ISIs of spiking to compute cross correlation here. The overall result, that asymmetry controls the phase of synchrony, is preserved. We apologize for any misdirection here.

13) Figure 8 legend: there is a typo: ‘from cell , the initially- cell, to cell .’ Cell IDs are missing.

Thanks, we have made these corrections.

14) (line 271-275) Reading these lines, it seems that asymmetry is not the general case in networks that authors quote, but authors deduce that ’asymmetrical location is a feature of coupling’.

Our intention in this section was only to point out that in the very few cases where GJs have been painstakingly localized, they have turned up in asymmetrical locations; and that locations of GJs vary widely, leaving much potential for asymmetric localization between cells. We have reorganized this section to be clearer about that intent:

Precise locations of electrical synapses along dendrites have proven difficult to exhaustively determine, but a handful of studies point towards asymmetrical localization. In coupled interneurons of cortex layer IV, synapses are located all along the dendrites, and measurements from 204 cells showed strong asymmetry in localization, with 90% synapses within 50 - 75 μm of one soma, but up to 250 μm away from the coupled soma (Fukuda, 2017). Asymmetrical localization also appears to be a feature of coupling between Golgi cells (Szoboszlay et al., 2016). The strongly asymmetrical synapses of the DCN also appear to couple mismatched distances from fusiform and stellate somas (Apostolides and Trussell, 2013). Other studies indicate that dendritic location of GJs is diverse across brain areas, and thus asymmetry could vary widely.

15) (line 281) ‘Dendritic integration is likely to be influenced by the presence of gap junctions along dendrites’ --this has been shown, e.g. by Vervaeke et al. (2012). The article should be quoted and added to the references.

Thanks, we have added this citation.

16) (line 289) idem, previous literature demonstrating this should be quoted.

Thanks, we have added citations there.

17) (line 291) ’Our results...’ is confusing and should be rewritten.

We have rewritten this sentence.

Reviewer #2

In this computational modeling study, the authors address the potential sources of the experimental observation of asymmetric electrical synapses and its consequences for circuit activity. Towards this, the authors first consider biologically plausible sources of asymmetry, focusing on intrinsic (morphology, compartment structure, and membrane resistance) and synaptic properties (coupling strength and location). Potential functional consequences are addressed, focusing on how spiking behavior can be altered by asymmetric electrical synapses.

The authors employed biophysical models and found that several distinct mechanisms can potentially give rise to asymmetric electrical synapses, which underscores the experimental challenge of pinpointing the precise biological mechanisms at play. This challenge is further highlighted by their demonstration of the difficulty of recovering a ground-truth asymmetric electrical synapse in even the simplest of scenarios (Figure 5). The authors go on to show that asymmetric electrical synapses can modify the spike latency and order of coupled bursters, which is extended to demonstrate the influence of asymmetric electrical synapses in the case of synchrony of tonic spikers.

This is a well written manuscript that systematically addresses the degeneracy of asymmetric electrical synapses in a way that would be experimentally infeasible. It provides the field with a framework for better understanding how electrical synapses contribute to neural circuit function. We have only minor issues that are listed below.

Thank you for the kind words.

Minor comments:

The simulations were mostly done with models of the same intrinsic properties except where explicitly specified. This is completely fine for the sake of demonstrating the points posed in the manuscript. However, it would be useful to the field to briefly discuss how these results would be influenced by heterogeneity in the intrinsic properties (ex. I_k, I_Ca, ... ) of the coupled cells.

Thanks for this addition. We have added text to the discussion (first PP) to include heterogeneity of ionic currents as another source of asymmetry.

Line 32: The first sentence of the introduction is very “mammalian” oriented, but the results clearly extend beyond GABAergic cells

Thanks. We have omitted the term GABAeric.

Line 34: Rectification is mentioned, but what is being considered here is not necessarily classical rectification as in a diode and as in the literature cited. In this manuscript, the asymmetry is modeled by unequal strength of gap junction conductance, and not rectification where current flows preferentially in a given direction.

Thank you for pointing out this distinction. We have removed the term rectification.

Line 38 stomatogastric ganglion instead of nucleus

Line 84: missing a period after the Mamiya reference

Thanks, we have made these corrections.

Line 99 or better Line 113 I missed a bit more info about the model (spiking/non spiking, LIF?) short description of properties, at least which currents are included.

Thanks, we have added text there to indicate that we used a HH model with a T-type calcium conductance in addition to leak, sodium and potassium.

Fig 1C which cell is cell1, not labeled. why headline D-D Synapse over voltage in C1 and C2 (also not specified in figure legend), as far as I understand it the current injection in C1 is into the soma and the colors represent the synapse location?. + it took me a while to figure out that the left voltage traces are cell 1 (I guess) and the right voltage traces are cell 2.

We’ve changed our labeling in Figure 1C, as well as the legend text for these subfigures to better represent cells, compartments, and traces.

May be worth including some equations in-line for clarity (ex. how asymmetry was modeled)

We added a general form of the Hodgkin Huxley equation used to model the cells and compartments in our experiments. We hope this will clarify how asymmetry was modelled (through altering coupling conductance for each cell), as well as other parameters.

Line 145/153 Is asymmetry defined by cc ratios? Line 152/153 talks about D-M synapses showed “lower values of asymmetry” meaning the cc ratio is closer to 1 than s-d. I have a problem with the term “lower values” for the cc ratios >1, (which is a bigger value in cc-ratio speak, but not in their use of “asymmetry”).

We agree that this was confusing. Here we are comparing effective asymmetry as measured by cc ratios. The wording ‘lower values’ has been changed to ‘lesser degree of effective asymmetry’ to reflect this.

Fig 2/3 their labeling some-middle synapse etc isn’t correct as they also show s-s and m-m etc. they are only limiting the possible synapse location to two of the three possibilities.

Thanks for noticing this, the labels have been removed

Fig 2 the two shades of orange/green are hard to distinguish.

We have increased the color ranges in Fig. 2 to better distinguish the data.

Line 217 ff/fig 7 again having troubling understanding what was done. Are both cells receiving bursting input? What does differing onset times mean? Fig 7a shows one set of voltage traces -> cell 1 or 2? Did they give the same bursting input in cell 1 and (?) in cell 2 time shifted and measured when the soma started spiking (which?)?

We added the text “to both cells” to clarify this point, and modified the next sentence to state explicitly that we varied the arrival (onset) times of the input bursts. We changed the legend text to state that Fig. 7A is the response of all 3 compartments in a single cell, and made other changes to simplify descriptions.

Fig 7D colors confusing as yellow and blue used in other contexts. Greyshades + neuron & synapse schematic would be helpful (instead of cell1, cell2 and connection only mentioned in figure legend). Figure legend refers to top, middle and bottom, figures labeled with 1, 2, 3.

Thanks - we made the suggested changes.

Line 308 what’s TC spike times?

We replaced TC with ‘thalamocortical cell’ there.

Line 224 refers to fig 7b, D-D, there’s no D-D in B,

We changed this to 7B.

Line 325ff very hard to figure out which value in line 327 and 328 belongs to currents listed in 325 ff

These sections have been re-written to clearly reflect the current and maximal conductance values used in our models.

Line 331 units are missing

We added units here.
